# Associations of Circulating Osteoglycin With Bone Parameters and Metabolic Markers in Patients With Diabetes

**DOI:** 10.3389/fendo.2021.649718

**Published:** 2021-03-15

**Authors:** Jakob Kau Starup-Linde, Rikke Viggers, Bente Langdahl, Soeren Gregersen, Simon Lykkeboe, Aase Handberg, Peter Vestergaard

**Affiliations:** ^1^ Department of Endocrinology and Internal Medicine, Aarhus University Hospital, Aarhus, Denmark; ^2^ Steno Diabetes Center Aarhus, Aarhus University Hospital, Aarhus, Denmark; ^3^ Steno Diabetes Center North Jutland, Aalborg University Hospital, Aalborg, Denmark; ^4^ Department of Endocrinology, Aalborg University Hospital, Aalborg, Denmark; ^5^ Department of Clinical Medicine, The Faculty of Medicine, Aalborg University, Aalborg, Denmark; ^6^ Department of Clinical Biochemistry, Aalborg University Hospital, Aalborg, Denmark

**Keywords:** bone, diabetes mellitus, osteoglycin, hyperglycemia, bone turnover

## Abstract

**Objective:**

Circulating osteoglycin may facilitate the crosstalk between bone and pancreas to empower adaptation of bone mass to whole body energy balance. We aimed to examine whether osteoglycin is associated with bone and metabolic parameters and if osteoglycin levels differ between patients with type 1 and 2 diabetes (T1D and T2D).

**Design and methods:**

A cross-sectional study of 190 patients with diabetes mellitus and stable hemoglobin A1c (HbA1c) (97 T1D and 93 T2D) was conducted. S-osteoglycin was analyzed by ELISA. Unpaired t-tests were performed to test differences between patients with T1D and T2D and linear regression analyses were performed to investigate associations between osteoglycin, glycemic markers, bone turnover markers and characteristics.

**Results:**

S-osteoglycin did not differ between patients with T1D and T2D (p=0.10). No associations were present between osteoglycin and age, gender, microvascular complications, HbA1c, or plasma glucose in T1D or T2D patients (p>0.05 for all). S-osteoglycin was not associated with levels of bone turnover markers (C-terminal cross-linked telopeptide of type-I collagen (CTX), P-procollagen type 1 amino terminal propeptide (P1NP), P-osteocalcin (OC), P-sclerostin, S-osteoprotegerin (OPG) or S-Receptor Activator of Nuclear factor Kappa beta Ligand (RANKL)) in neither T1D or T2D patients (p>0.05 for all).

**Conclusion:**

Osteoglycin levels were similar in T1D and T2D patients. Osteoglycin did not correlate with glucose, HbA1c or any other biochemical marker of bone turnover. Thus, we did not find evidence supporting the existence of an osteoglycin-bone-pancreas axis.

**Clinical Trial Registration:**

ClinicalTrials.gov, identifier NCT01870557.

## Introduction

Diabetes mellitus (DM) is a chronic metabolic derangement often leading to serious complications that may affect multiple organs (1). Recently, it has become evident that diabetes is associated with increased risk of fractures that cannot be explained by reduced areal bone mineral density (aBMD) (2, 3).

Bone turnover is reported to be lower in patients with T2D compared to T1D (4, 5). Markers of bone turnover has expanded over the last decade and include a number of suggested potential markers to be used in a clinical setting in addition to aBMD (6).

Osteoglycin is a proteoglycan rich in leucine which derives from bone, cartilage and myocytes, and can be detected in serum (7–9). Osteoglycin enhance bone mineralization and formation by stimulating alkaline phosphatase and osteocalcin mRNA in osteoblasts examined *in vitro* (6, 10). Furthermore, osteoglycin expression by myoblast is increased by addition of active 1,25 Vitamin D (10, 11). Osteoglycin is hypothesized to have endocrine effects on bone and pancreas. Osteoglycin knockout mice displayed increasing amounts of white adipose tissue and impaired glucose tolerance independent of diet consumption (12). Another study reported higher femoral bone mineral content and abnormal increased size of collagen fibrils in osteoglycin deficient mice compared to wild type littermates (13). The increased bone mass in osteoglycin deficient mice suggest osteoglycin to reduce osteoblast activity by down-stream mediation of Y1 receptor signaling, resulting in an increased number of osteoclasts and impaired mineralization (12). Also, treatment with osteoglycin in mice was related to lowering of blood glucose in a dose dependent manner as well as potentiating lowering of glucose during insulin tolerance test (12).

Human studies investigating osteoglycin are sparse. Osteoglycin was more abundant in visceral adipose tissue compared to white adipose tissue in overweight human subjects (14). In postmenopausal women with T2D, osteoglycin levels are associated with duration of diabetes, decreased aBMD and presence of vertebral fractures but not N-terminal cross-linked telopeptide of type-I collagen (NTX), osteocalcin (OC), fasting glucose or HbA1c (15). These findings suggest osteoglycin to be a marker of low aBMD and vertebral fractures in T2D.

Examination of S-osteoglycin in humans following gastric surgery showed negative correlation with body mass index (BMI) and positive correlation with lean body mass as well as increasing levels of S-osteoglycin during weight loss (12). However, gastric surgery itself may impact bone health and metabolic markers in T2D by other mechanisms than osteoglycin (16, 17).

Based on the current evidence, osteoglycin may be a metabolically active molecule and impact on insulin action and glucose homeostasis.

Regarding current knowledge, we wondered if osteoglycin levels depends on pancreas function as T1D is characterized by insulin depletion whereas T2D is a state of insulin resistance and initially hyperinsulinemia. We hypothesized that osteoglycin levels were not different between patients with T1D and T2D. We aimed to examine osteoglycin levels in patients with T1D and T2D and investigate potential associations between osteoglycin and bone turnover markers, bone density measures, bone structural markers and metabolic parameters in respect to pancreas dysfunction and insulin resistance.

## Methods

This cross-sectional study utilizes blood samples and bone scans from previously published studies (4, 18). The STROBE statement guideline for reports of cross-sectional studies was followed (19). The trial was registered at ClinicalTrials.gov (NCT01870557).

### Ethical Approval

The study protocol was approved by the Ethics Committee of the Central Denmark Region (registration number: 1-10-72-5-13) and follows the Declaration of Helsinki II. Informed consent was obtained from all individuals in the trial.

### Study Design

In the original study we included 200 participants and 197 were examined. Three participants dropped out due to lack of time. We excluded one participant in bisphosphonate treatment, two pre-menopausal women, one patient with unmeasurable high osteoglycin levels and three patients where measurements were not obtained.190 patients with physician diagnosed diabetes mellitus (97 T1D and 93 T2D) were consecutively included from 2013-2014 at Aalborg (n=74) and Aarhus University Hospitals (n=116) in a cross-sectional study. The physician diagnosis of T1D is supported by measurement of antibodies if there is uncertainty of diabetes type. All patients were > 50 years of age and had a relatively stable HbA1c within the last six months (± 1% based on the DCCT scale). Furthermore, all patients had a recent estimated glomerular filtration rate (eGFR) above 50 ml/minute. The participants were not on weight loss- or ketogenic diets. Physical activity was assessed by a questionnaire that has previously been used to assess if physical activity is held constant over time (20–23). Exclusion criteria were kidney disease defined by eGFR below 50 ml/minute or macro albuminuria, hyperthyroidism and hypothyroidism (treated hypothyroidism with TSH within reference range were included), NYHA class IV heart failure, and diseases or conditions affecting bone. Potential participants treated with antiepileptics, glucocorticoids, lithium, bone antiresorptive treatment, estrogen treatment, and bone anabolic treatment were excluded. Pre-menopausal women were excluded from the study. Information about diabetes related complications, medication use, and lifestyle factors were collected at baseline.

### Blood Sample Analysis

Blood samples were collected in non-fasting conditions in the morning (generally before 12 pm). Osteoglycin was analyzed by the Enzyme-linked Immunosorbent Assay Kit (Human, SEC688Hu) from Cloud-Clone Corp. (Katy, Tx, USA) as described by the manufacturer. In brief, serum samples were diluted 17.7 fold in 0.01 mol/L phosphate buffered saline (PBS), pH 7.2 (Dulbecco PBS, Gibco # 14190-094) and analyzed in duplicates. According to the manufacturer, no significant cross-reactivity or interference between osteoglycin and analogues have been observed, and the intra-assay coefficient of variation (CV) was < 10%, and inter-assay CV was < 12%. In our study, intra-assay CV was 11.4% (N=196 serum duplicates), and inter-assay was 17% (osteoglycin quality control) and 12.6% (serum aliquot). In three subjects, the samples were missing or analysis could not be conducted and one subject had immeasurable high levels and was excluded from the analysis. The subject with immeasurable high levels of osteoglycin did not differ from the other participants in pharmaceutical use, age, BMI, glycemic markers (p-glucose and HbA1c), and bone turnover markers. P-C-terminal cross-linked telopeptide of type-I collagen (CTX), P-OC, and P-procollagen type 1 amino terminal propeptide (P1NP) were measured as single determinations on an automated Cobas analyzer from Roche Diagnostics (Mannheim, Germany). P-sclerostin/S-OPG (Biomedica, Vienna, Austria) and free non-OPG-bound S-RANKL (Biomedica, Vienna, Austria) were measured as ELISA double determinations. The analytical coefficients of variation were according to the manufacturers as follows: CTX < 6%, P1NP < 4%, OC < 2%, < 5%, Sclerostin < 10%, RANKL < 3%, OPG < 5%. All bone turnover markers were analyzed on EDTA-plasma, except for RANKL and osteoprotegerin (OPG) which were measured on serum. Measurements were performed in clinical biochemical laboratories accredited according to ISO 15189.

### DXA and HRpQCT Scans

The scanning techniques have been described in detail elsewhere (18). Trained staff performed dual energy x-ray absorptiometry (DXA) measuring aBMD at the hip and lumbar spine. Two Hologic Discovery scanners were used at Aarhus University Hospital and two Lunar Prodigy scanners were used at Aalborg University Hospital. The intra scanner precision CVs were 1% for both Hologic Discovery and Lunar Prodigy scanners.

Patients at Aarhus University Hospital were examined by the quantitative computed tomography (HRpQCT) scan (Xtreme CT, Scanco Medical, Switzerland) at the radius and tibia. A standard operating procedure was followed. Standard evaluation analysis, finite element analysis, and cortical evaluation were performed. To ensure reproducibility all scans and evaluations were performed by J. Starup-Linde. The precision CVs were 0.7% and 1% for the tibia and radius, respectively.

### Data on Fractures

Fracture diagnoses were extracted from the Danish National Hospital Discharge Register in the time period January 1, 1977 to March 10, 2015. The registry was founded in 1977 and covers all inpatient contacts from 1977 to 1994 and from 1995 also all outpatient visits to hospitals, outpatient clinics, and emergency rooms. Fractures were grouped as previous fractures and incident fractures subsequent to the study. Vertebral fractures were assessed by Vertebral Fracture Assessment (VFA) and x-ray was performed for confirmation at Aarhus University Hospital and by x-ray of thoracic and lumbar spine at Aalborg University Hospital.

### Statistical Analysis

Descriptive statistics were used to present patient characteristics and the relation to biochemical bone turnover markers. STATA 8 was used to perform the analyses. Mean values of previously measured HbA1c of the last five years were calculated as the time span between the first available measurements to the date of the study blood sample. The mean HbA1c was used to explore whether long-term glycemic control was associated with levels of osteoglycin.

Unpaired t-test was performed to test for differences between patients with T1D and T2D and linear regression analyses were performed to detect associations between osteoglycin and glycemic markers, bone turnover markers, and subject characteristics. Bartlett’s test was used to determine if the t-test should be performed with equal or unequal variances. Assumptions were checked. Logistic regression analysis was performed to investigate associations between osteoglycin and fractures.

The *a priori* hypothesis was that osteoglycin is associated with bone turnover in patients with diabetes. A *post hoc* power calculation would be highly dependent on the current study results and this may not be valid, however we performed a posthoc power calculation based on expected differences in patients with T1D and T2D. We expected a difference of 500 ng/ml osteoglycin between T1D and T2D and assumed a sd of 500 thus 44 participants are needed.

## Results

The study presents a post-hoc analysis from a previously published study (4, 18).

### Characteristics

**Table 1** presents the participant characteristics. We report data on 97 patients with T1D and 93 patients with T2D. Patients with T1D were younger, had a lower BMI and longer diabetes duration. The HbA1c of the participants ranged between 39 and 106 (mean 64 ± 9 mmol/mol) with no differences between patients with T1D or T2D. Vitamin D levels were significantly lower in patients with T2D (64.6 nmol/l vs. 74.2 nmol/l), however for both groups the mean vitamin D levels were within normal range. Also, bone formative markers (P-P1NP and P-OC) were lower in patients with T1D. Data on physical activity were missing in some subjects. We detected a lower level of daily life physical activity in participants with T2D compared to T1D, whereas there were no differences in physical activity during sports and at work. Data on bone turnover marker, bone structure and fracture differences between T1D and T2D have previously been presented (4, 18).

**Table 1:** Characteristics of the included subjects.

	Type 1 diabetes (n=97)	Type 2 diabetes (n=93)
**Characteristics**	mean (95% CI)	mean (95% CI)
Age (years)^α^	60.8 (59.3;62.3)	65.2 (63.6;66.8)
BMI (kg/m^2^)^α^	25.8 (25.0;26.6)	30.7 (29.9;31.6)
Gender (male %)	60 (50;70)	63 (53;73)
Diabetes duration (years)*^α^	24.3 (21.7;26.8)	14.5 (12.9;16.1)
Microvascular complication (%)	48 (38;59)	46 (36;57)
Current smoker (%)	19 (11;26)	30 (21;40)
Alcohol intake (units/week)^α^	7 (5.5;8.5)	4.8 (3.4;6.2)
Insulin users (%)^α^	100	0.60 (0.50;0.70)
**Biochemical parameters**		
Osteoglycin (ng/ml)	6.92 (6.47;7.37)	7.42 (7.01;7.83)
CTX (ng/ml)	0.22 (0.20;0.24)	0.19 (0.17;0.21)
Osteocalcin^α^ (ng/ml)	19.7 (18.4;20.9)	15.6 (14.4;16.9)
P1NP^α^ (ng/ml)	41.3 (38.4;44.4)	36.0 (33.0;39.1)
Creatinine (mmol/l)	75.0 (71.7;78.3)	76.1 (72.3;79.9)
Vitamin D^α^ (nmol/l)	74.2 (67.6;80.9)	64.6 (58.3;70.9)
HbA1c (mmol/mol)	64.7 (62.8;66.6)	63.5 (61.5;65.4)
Mean HbA1c of last five years (mmol/mol)	66.3 (64.6;67.9)	64.5 (62.9;66.1)
Random p-glucose* (mmol/l)	10.7 (9.71;11.6)	10.9 (10.1;11.7)
**Physical activity of ½ hour (non-sports fx gardening) (n=175)** ^α^		
≥ 4 times per week (n)	34	30
2-3 times per week (n)	39	32
1 time per week (n)	11	8
2-3 times per month (n)	5	8
Less frequently (n)	5	7
**Physical activity in sports of ½ hour (n=167)**		
≥ 4 times per week (n)	9	10
2-3 times per week (n)	30	20
1 time per week (n)	22	16
2-3 times per month (n)	6	6
Less frequently (n)	18	30
**Physical activity at work (n=162)**		
Office work or unemployed (n)	59	61
Some physical activity at work but no heavy lifts (n)	8	8
Moderate physical activity at work with heavy lifts (n)	13	9
Very physical active work with heavy lifts (n)	5	5

^α^p < 0.05 between T1D and T2D *t-test with unequal variances due to Bartlett’s test. If n is less than 5 for physical activity measures 5 is reported.

### Measurement of S-Osteoglycin and Associations With Metabolic Markers

S-osteoglycin levels were not different between patients with T1D and T2D (p=0.10). S-osteoglycin levels were positively associated with BMI for the entire population, r=0.081 (95% CI 0.017; 0.144), however the association became insignificant in sensitivity analyses for T1D and T2D, respectively. Age, gender, diabetes duration, microvascular complications, smoking, alcohol use and insulin use were not associated with S-osteoglycin levels. **Table 2** presents the results of the linear regression analysis between osteoglycin and metabolic markers. Results for osteocalcin have previously been published, and osteocalcin showed significant associations with metabolic markers (as plasma glucose and HbA1c) and bone turnover markers (as CTX and P1NP) which underline that the present study is powered to detect associations (4, 18). Osteoglycin was not associated with the current HbA1c level, mean HbA1c of previous 5 years, or random plasma glucose level in neither patients with T1D nor T2D (p>0.05 for all) and neither in analyses adjusted for age, BMI and gender. In sensitivity analyses, similar results were revealed for patients with T1D and T2D. The association between osteoglycin levels and the use of oral antidiabetics, insulin and glucagon-like peptide-1 receptor agonists (GLP-1) was investigated. Neither SU (23 users), insulin (56 users) metformin (72 users) nor SGLT-2inhibitors (4 users) were associated with osteoglycin levels (p>0.05 for all). Use of GLP-1 receptor agonist (36 users) were associated with higher levels of osteoglycin (8026, 95% CI 7262-8788 vs. 7041, 95% CI 6583-7500, p=0.0198) whereas use of DPP-IV inhibitors (11 users) were associated with lower levels of osteoglycin (7572, 95% CI 7125-8018vs. 6303, 95% CI 5445-7161, p=0.0472). We observed no association between osteoglycin levels and measures of self-reported physical activity.

**Table 2:** Regression analysis of osteoglycin with bone and metabolic parameters.

Variable	s-Osteoglycin (coefficient and 95% CI)
**Metabolic parameters**	
HbA1c (mmol/mol)	0.010 (-0.021;0.043)
Mean HbA1c of last five years (mmol/mol)	0.028 (-0.009;0.066)
Random p-glucose (mmol/l)	-0.003 (-0.075;0.068)
**Bone turnover and signaling markers**	
P-CTX (ng/ml)	-0.234 (-3.11;2.65)
P-Osteocalcin (ng/ml)	-0.034 (-0.082;0.014)
P-P1NP (ng/ml)	-0.008 (-0.029;0.012)
P-Sclerostin (pmol/l)	-0.001 (-0.010;0.010)
S-RANKL (pmol/l)	-2.47(-7.61;2.67)
S-Osteoprotegerin (pmol/l)	0.036 (-0.107;0.180)
**Bone density and structural markers**	
aBMD of the hip (n=187) (g/cm^2^)	1.041 (-0.991;3.003)
aBMD of the femur (n=187) (g/cm^2^)	0.442 (-1.850;2.735)
aBMD of the lumbar spine (n=189) (g/cm^2^)	0.429 (-1.303;2.162)
aBMD of the distal forearm (n=189) (g/cm^2^)	1.231 (-1.990;4.454)
Failure load radius (kN)	-0.001 (-0.001;0.001)
Failure load tibia (kN)	0.001 (-0.001;0.001)
vBMD radius (n=111) (mg HA/cm^3^)	-0.002 (-0.008;0.005)
vBMD tibia (n=113) (mg HA/cm^3^)	-0.001 (-0.008;0.008)

### Association of Osteoglycin With Bone Markers

**Figure 1:**
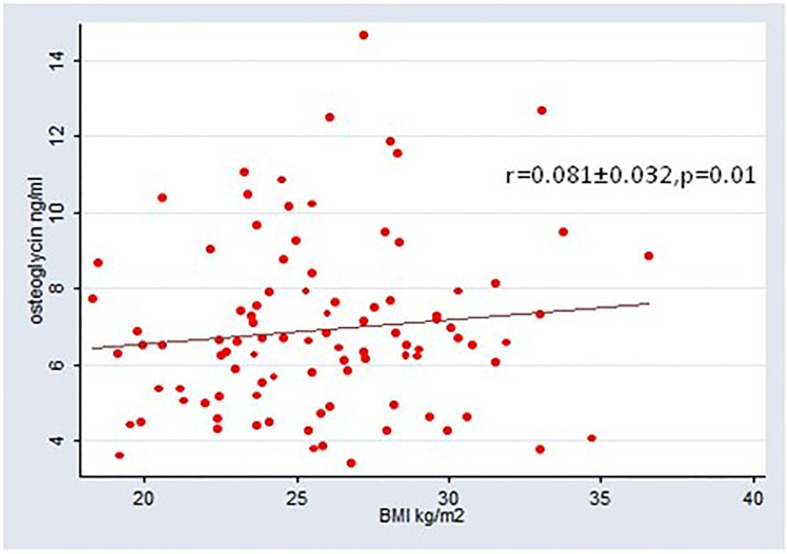
Association between BMI and osteoglycin.

**Table 2** presents the results of the linear regression analysis between S-osteoglycin, bone turnover markers, bone density and structural markers. S-osteoglycin was not associated with the bone turnover markers P-CTX, P-P1NP, P-OC, P-sclerostin, S-OPG or S-RANKL levels and neither in analyses adjusted for age, BMI and gender. S-osteoglycin levels were not associated with aBMD at the hip, femur, lumbar spine or distal forearm. HRpQCT analyses of the bone structure revealed no association with S-osteoglycin levels neither for trabecular markers nor cortical markers. In sensitivity analyses, similar results were revealed for patients with T1D and T2D. **Figure 1** displays a positive and significant association between S-osteoglycin and BMI. We did not find any significant correlations between S-osteoglycin and HbA1c (data not shown). Multivariate analyses did not change any results.

### Association With Prevalent Vertebral Fractures and Incident Fractures

No associations between S-osteoglycin levels and prevalent vertebral fractures, incident fractures, or previous fractures was demonstrated using logistic regression analyses (p>0.05 for all).

## Discussion

To our knowledge, this study is the first to report on S-osteoglycin levels in patients with T1D. We found no difference in S-osteoglycin levels between patients with T1D and T2D. We found no evidence of an association between S-osteoglycin and bone turnover markers, bone mineral density, bone structural markers or metabolic markers. Furthermore, we could not confirm an association between S-osteoglycin and vertebral fractures. The association between BMI and S-osteoglycin for the full group of participants may reflect that BMI and type 2 diabetes are linear covariates and we found no association between BMI and S-osteoglycin in sensitivity analyses of patients with T1D or T2D. Osteoglycin is hypothesized to be a novel marker of a muscle, pancreas, and bone axis. This study includes diabetes patients presumably with and without pancreatic dysfunction (i.e. T1D and T2D) and so; we do not support the hypothesis.

Our study includes a large well-characterized population of patients with T1D and T2D that contribute strength to the results. The sample size made it possible to compare diabetes types and adjust for relevant variables. We find no clear effects of glucose-lowering drugs on osteoglycin levels. We suspect that the opposite associations of DPP-IV and GLP-1 on osteoglycin levels is a chance finding, although GLP-1 infusion has been shown to recruit skeletal muscle in humans (24).

Blood samples were collected in a non-fasting state that may affect serum levels of osteoglycin. P-P1NP and P-OC are relatively stable markers independent of time of day or fasting status, whereas P-CTX follows a circadian rhythm and is suppressed by food intake (25, 26). A non-fasting state may influence the variability of S-osteoglycin levels. In addition, it is still unknown, whether the transcription and/or secretion of osteoglycin exhibit a circadian rhythm. Thus, a limitation of the presented results is the possible importance of sample collection timing that may explain the insignificant results. Diabetes mellitus is characterized as a disease with a disrupted fasting state (27), and thus, even in fasting conditions diabetes patients may present with elevated P-glucose levels. However, there are no published studies on the effect of fasting versus non-fasting state on S-osteoglycin levels in humans and neither before or after insulin or glucose administration. Therefore, data on S-osteoglycin levels before and after an oral glucose tolerance test or meal as well as over time could provide further information.

The expression of osteoglycin is previously reported higher in visceral adipose tissue samples compared to subcutaneous adipose tissue and in particular, high in obese subjects compared to lean subject (14). This supports our finding of a positive association between BMI and osteoglycin. Another study found osteoglycin to be negatively correlated with body fat in mice and BMI in humans (12). However, this was limited by 19 subjects that all were severely obese. In addition, the same study found increased white adipose tissue fat mass and respiratory quotient in osteoglycin knockout mice indicating metabolic flexibility and shift toward glucose oxidation. During fasting and insulin infusion, the respiratory quotient has been reported lower in healthy obese humans compared to lean humans (28). Collectively, data on the possible association between osteoglycin and BMI/fat mass in humans and rodent seem conflicting. To reveal if any association occur, we propose future research to measure osteoglycin in adipose tissue biopsies as well as blood samples from lean untreated and obese subjects in the fasting state.

The participants with T1D had a 10 year longer diabetes duration compared to participants with T2D, however in T2D the diagnosis seems delayed by 4-6 years (29). In the present study we found no association between osteoglycin levels and diabetes duration in neither patients with T1D or T2D. With longer diabetes duration there may be an accumulation of AGE products that is hypothesized to cause bone fragility (30). Bone turnover markers are also decreased in the pre-diabetic state (31), and it is unknown whether longer diabetes duration independent of glycemic levels reduce bone turnover marker levels further.

S-osteoglycin levels have previously been associated with fractures and low aBMD in patients with T2D (15), however this study could not confirm these findings. This study used similar assays as used in our study. In comparison the mentioned study was performed on Japanese subjects with a slightly older age, higher HbA1c and lower BMI compared to the T2D subjects included in the present study (15).

Although mice with osteoglycin knockout display altered metabolic and bone profile suggesting a bone-muscle- and pancreas-axis (12, 13), we could not find any association between osteoglycin and these markers in humans with diabetes. This may be due to difference between mice and humans, or the alterations observed in the bone and metabolic profile may only occur at very low osteoglycin levels. Knowledge about the dynamics and metabolization of osteoglycin are sparse. Moreover, there may be counter-regulatory mechanisms to low S-osteoglycin levels in humans which are not identified such as the circadian rhythm, feeding, and exercise. In vitro and animal models are limited, and very few studies have reported on osteoglycin in humans.

A bone pancreas axis has previously been proposed for OC, a product of the osteoblasts, and especially its undercarboxylated OC (ucOC) form. Based on mice models ucOC is suggested to increase insulin production and insulin release from pancreatic β-cells and conversely insulin is suggested to increase the production of ucOC (32–34). However, in humans the evidence of an effect of ucOC axis on glucose metabolism and insulin release is very limited as an increase in ucOC by parathyroid hormone treatment did not affect markers of energy metabolism (35). Neither were there any difference in ucOC by an oral glucose tolerance test or intravenous isoglycemic glucose infusion despite differential insulin responses (36). The discrepancy between the results of mice models and human studies for ucOC highlights that not all finding in mice may translate into humans, which also may be the case for osteoglycin.

The present study is limited by its cross-sectional nature. The study cannot conclude on causality. Furthermore, we have no healthy comparison group to neither T1D nor T2D. Moreover, no reference range for S-osteoglycin levels in humans is known. Thus, we cannot conclude whether the levels observed are high or low. The current study was performed in a group of patients with diabetes. Further perspectives would be to compare osteoglycin between patients with diabetes and non-diabetic controls. However, due to the relationship between muscle mass and osteoglycin such a study would need to correct for muscle mass measured by sat whole body DXA and/or muscle strength by say handgrip strength, but we observed no association between osteoglycin levels and self-reported physical activity. Trabecular bone score may improve fracture detection in patients with T2D (37) and may be associated with osteoglycin. However, we found no association between osteoglycin and other bone structural measures in this study. Furthermore, we had no data on 1,25 hydroxy vitamin D, but used 25 hydroxy vitamin D as a surrogate marker as 1,25(OH)2D may be a biased marker of true vitamin D status.

Impaired glucose metabolism is associated with compromised bone integrity and the existence of increased risk of fractures among patients with diabetes is increasingly recognized as an important complication of both T1D and T2D and the risk seems to increase with poor glycemic control (3, 38–41). Positive energy balance paves the way of increase in body weight. To prevent fractures, it is pivotal that the skeleton empowers the ability to adjust by increasing its strength to match the higher mechanical demand. Osteoglycin has been hypothesized to influence both bone and metabolic markers and could be an important marker or mediator of metabolic health, though the current study does not support this hypothesis. However, current evidence on S-osteoglycin levels in humans is limited and further research is needed. There is a consensus on the fact that osteoglycin is expressed and secreted from muscle cells and future studies could focus on S-osteoglycin levels in human subjects with or without diabetes mellitus as well as before and after exercise.

In conclusion, we did not find evidence supporting the existence of an osteoglycin-bone-pancreas axis in humans with diabetes. In this cross-sectional trial S-osteoglycin levels were similar in T1D and T2D patients and did not correlate with glucose, HbA1c or any other biochemical marker of bone turnover.

## Data Availability Statement

The raw data supporting the conclusions of this article will be made available by the authors, without undue reservation.

## Ethics Statement

The studies involving human participants were reviewed and approved by The Ethics Committee of the Central Denmark Region (registration number: 1-10-72-5-13). Follows the Declaration of Helsinki II. The patients/participants provided their written informed consent to participate in this study.

## Author Contributions

RV and JS-L contributed with conception, ideas, analysis interpretation, primary drafting as well as several critical revisions of text, tables and figures and finalization. SL and AH took part in biochemical analyses and interpretation of data as well as critical revisions. BL, SG and PV took part in funding and critical revision of conception, analysis and interpretation. All authors contributed to the article and approved the submitted version.

## Funding

This work was supported by Steno Collaborative grant, Novo Nordisk Foundation, Denmark (Grant no. NNF18OC0052064).

## Conflict of Interest

The authors declare that the research was conducted in the absence of any commercial or financial relationships that could be construed as a potential conflict of interest.
